# Unveiling gender dynamics and disparities in the aquaculture value chain: evidence from Ogun and Delta States, Nigeria

**DOI:** 10.1007/s10499-025-01966-1

**Published:** 2025-05-16

**Authors:** Rahma Isaack Adam, Lucy G. Njogu, Kevin Okoth Ouko, Surendran Rajaratnam, Lydia Adeleke, Lydia Ogunya, Elizabeth Ihiechi Akuwa, Cathy Razel Farnworth, Bernadette Fregene

**Affiliations:** 1 WorldFish Kenya, C/O International Livestock Research Institute, Nairobi, 00100 Kenya; 2 Center for Research in Psychology and Human Well-Being, Faculty of Social Sciences & Humanities (FSSK), Universiti Kebangsaan Malaysia, 43600 Bangi, Selangor Darul Ehsan Malaysia; 3 Federal University of Technology Akure, Akure, Ondo State Nigeria; 4 International Institute of Tropical Agriculture (IITA) Headquarters, PMB 5320, Oyo Road, Ibadan, 20001 Oyo State Nigeria; 5 WorldFish Nigeria, C/O IITA Headquarters, PMB 5320, Oyo Road, Ibadan, 20001 Oyo State Nigeria; 6 Pandia Consulting, Teigelkamp 64, 48145 Münster, Germany; 7 School of Global Development, University of East Anglia, Norwich, UK

**Keywords:** Gender roles, Gender relations, Participation, Aquaculture value chain, Nigeria

## Abstract

This paper offers new insights into gender norms, roles, participation, relations, and benefits derived by women and men engaged in the aquaculture sector in Ogun and Delta States in Nigeria. Data were collected using mixed methods, including structured surveys of 410 farmers, 175 market actors, and 53 input suppliers, 116 semi-structured key informant interviews, and 11 focus group discussions (FGDs). Overall results of the study revealed the linkages within the aquaculture value chain, which was highly gendered, with men dominating all the three main stages of the value chain as indicated by Duncan’s index of dissimilarity of 17.35%. Results also revealed a gender difference in the value of assets, ownership, and wage rate among men and women participants in paid labor in the input supply and fish trading segments. Men tended to realize more profits than women, indicating an imbalance in the distribution of benefits by gender along the aquaculture value chain. Results revealed that the participation of women in decision-making was relatively high, attributable to their involvement in aquaculture value chain activities. The findings highlight the need for governments, development agencies, and non-governmental organizations to address gender disparities in policies designed to improve the imbalance in the distribution of benefits between women and men.

## Introduction

Despite its current small contribution to global aquaculture production (FAO 2022), Africa has enjoyed remarkable growth in the aquaculture industry over the past decade owing to improved food systems policies, targeted investment in research and training, increased private sector investment, and enhanced financing (Tran et al. 2019). Being the second-largest aquaculture producer (11.12% in 2022) after Egypt (67.62% in 2022) (FAO 2022), and the largest fish consumer in the continent, Nigeria is important to Africa’s aquaculture production and growth (Adeleke et al. 2020).

However, Nigeria experiences a major disparity between its domestic consumption requirements and production levels. Annual production is around 300,000 tons while consumption totals approximately 3.2 million tons (Emmanuel et al. 2014; Issa et al. 2022). In itself, this disparity calls for a better understanding of the opportunities and challenges in Nigeria’s aquaculture value chains.^1^

A holistic approach to value chain analysis focuses on three aspects of sustainability: economic, environmental—encompassing biological and technical components, and social (Krause et al. 2020). Mostly, aquaculture interventions focus primarily on strengthening the sector’s economic and technical components, paying less attention to social sustainability (Adam et al. 2021; Omeje et al. 2021). However, the United Nations Sustainable Development Goals (SDGs) emphasize that achieving social sustainability and gender equality (SDG 5) is integral to the ability of value chains to deliver food and nutrition security. This is formulated in SDG 2: “End hunger, achieve food security and improved nutrition, and promote sustainable agriculture,” and SDG 14: “Life below Water” similarly highlights the importance of working sustainably across aquatic food systems (UN DESA 2023; Kruijssen et al. 2018). We are, therefore, tasked with bringing together the three pillars of sustainability on an equal footing. In relation to gender equality and women’s empowerment (GEWE) specifically, a gender and social justice approach insists that women and men have an inherent equal entitlement to gain from development (Nussbaum 2003; Sen 1995). Turning to an instrumental approach to development, empirical research suggests that improved GEWE aims to achieve better food security and nutrition and secure broader improvements in development outcomes (Leal Filho et al. 2022; Cole et al. 2015; Weeratunge et al. 2010; Shailaja & Madeleine 2008). These arguments have been made for decades by social scientists, and based on empirical evidence are increasingly influential in development cooperation. Increasingly, both approaches are seen as mutually intrinsic and synergistic. The German government, for example, is implementing a feminist development policy which argues that gender equality is central to the creation of socially just, economically, and ecologically sustainable democratic societies (BMZ 2023). Private sector players are increasingly recognizing that gender inequalities contribute directly to market inefficiencies (Kruijssen et al. 2018). Fair Trade businesses implement the assumption that “business, trade, and the economy can be redistributive and regenerative by design” (Kiessel, 2022: 29) and they seek to combine economically profitable value chains with social and gender justice (Farnworth et al. 2016).

Normative change in gender norms is increasingly seen as a pre-requisite to the achievement of GEWE and associated desirable development outcomes (FAO et al. 2022). Gender norms are a subset of social norms. They define acceptable and appropriate actions for women and men in a given group or society (Cislaghi and Heise 2020). Gender norms are dynamic and vary across time (Rolleri 2013), and differ across groups and communities, and may be specific to particular social groups within a community (Rietveld et al. 2023; Cislaghi and Heise 2020; Rolleri 2013). Moreover, gender norms and dynamics are expressions of power relations. They create and reproduce systemic differences in the positions of different groups of people, with gender expressing specific forms of power relations between women and men (Kruijssen et al. 2022). Gendered power relations contribute to gendered variations in health and educational outcomes; shape the division and allocation of labor between productive, social, and reproductive (household and care) tasks; influence women’s and men’s access to and control over productive resources; guide the efficacy of voice in decision-making processes and help to determine gendered rights and privileges at all levels (Idiku et al. 2022; Mwongera et al. 2017). Gender norms are not necessarily negative, but discriminatory or harmful gender norms hamper women’s ability to develop their agency and capabilities, and therefore reduce the ability of women to empower themselves (FAO et al. 2022; Christopherson et al. 2022; Marcus 2021; Idris, 2018). Indeed, gender norms may serve to obscure women as economic actors because they do not appear to own the means of production (Kruijssen et al. 2018). Yet although men may benefit from privileged access to resources and power, they may also be harmed through having to live up to gender-inequitable masculinities (OECD 2021).

Gender-inclusive value chains ensure that women participate in and benefit equally with men in value chains. It involves ensuring that women are included across various nodes of the value chain, that they have the material and social resources they require to be effective actors, and that they can secure benefits commensurate to their efforts (Gopal et al. 2020; Ramirez et al. 2020; Githukia et al. 2020; Kruijssen et al. 2018).

A systematic review (Adam and Njogi, 2023) of 78 articles on gender and aquaculture in Nigeria found considerable gender inequalities. Women are located in less profitable and less resource-intensive nodes of the value chain—generally processing and retailing, whereas men concentrate on fingerling production and grow-out fish farming. Women typically depend on spouses for investment capital. Due to their frequently weak bargaining power, this can restrict women’s power and agency. Formal sources of credit are mostly closed to women due to high interest rates, collateral requirements, and widespread financial illiteracy. More broadly, as their findings revealed, there is a significant lack of sex-disaggregated data which hampers the ability of development actors to improve aquaculture value chains.

This paper contributes to filling these knowledge gaps by mapping gender roles, their levels of engagement, and benefits along the Nigerian aquaculture value chain through a gendered aquaculture value chain analysis framework developed by Kruijssen et al. (2021). It aims to highlight the challenges and opportunities available for men and women in the aquaculture sector in the country. Empirical qualitative and quantitative data from a study conducted in two states in Nigeria in 2022 is presented and analyzed. Development actors including governments, private sector, and development partners are expected to benefit through acquiring insights into how aquaculture interventions may interface with gender norms and dynamics. A core assumption underlying the research is that improving women’s access in aquaculture value chains without increasing their work burden requires a holistic, critical, and coordinated approach to understanding these value chains and identifying bottlenecks and opportunities for women to participate in aquaculture commodities’ production, processing, and marketing.

This paper is divided into six sections. Following this section, we explain our “Materials and methods,” followed by the “Results,” the “Discussion,” and “Conclusion.” We end the paper with the “Limitations of the study.”

## Materials and methods

### Conceptual framework

**Fig. 1:**
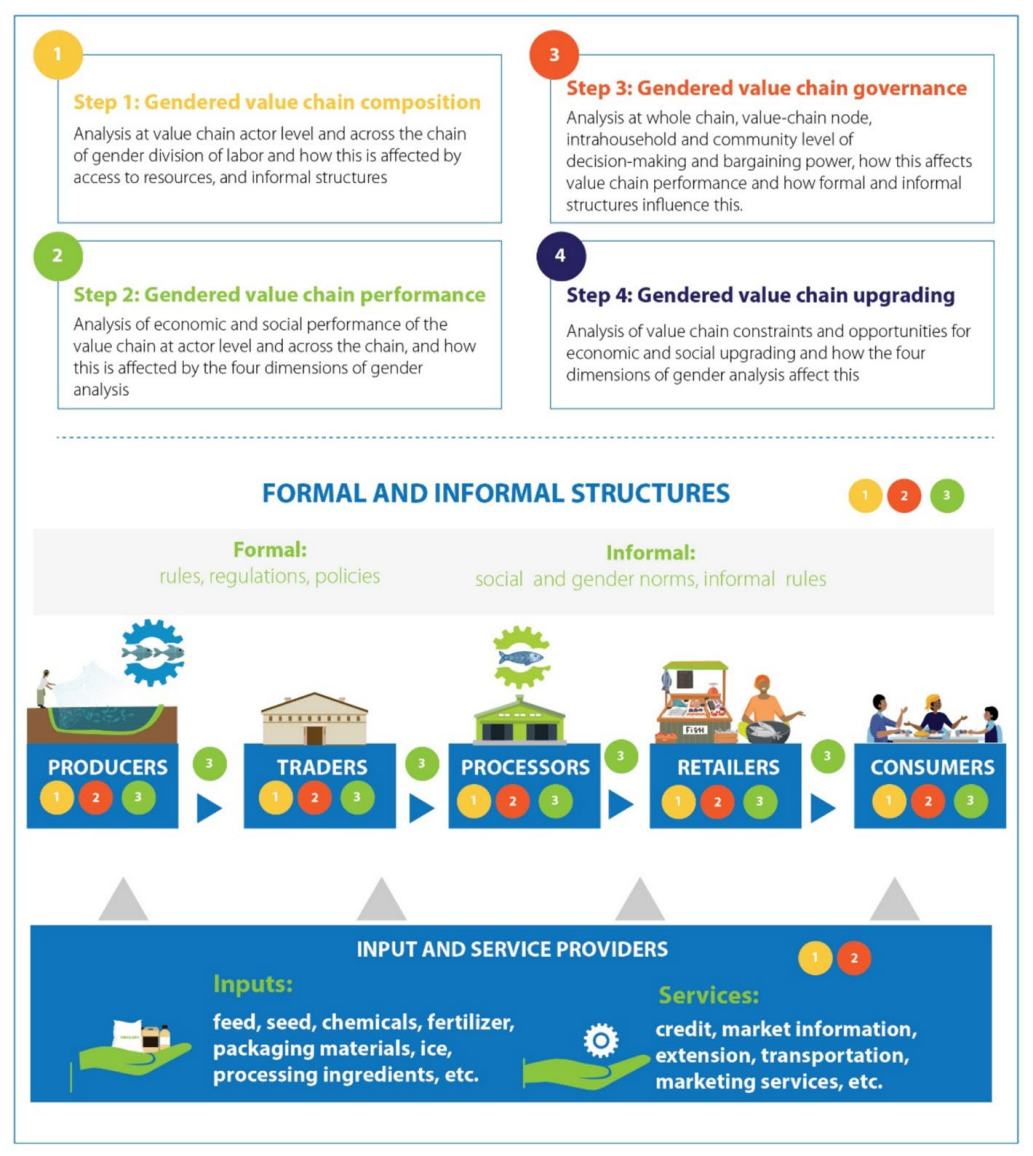
Steps and entry points to apply a gendered aquaculture value chain analysis (source: Kruijssen et al. 2021)

We used the gendered aquaculture value chain framework (Kruijssen et al. 2021). This framework facilitates an understanding of the underlying gender norms and dynamics which create gender inequalities. It outlines important analytical principles and parameters, incorporates a variety of gender outcomes, and provides an analytic framework for interpreting empirical data in ways which expediate the development of recommendations for gender-equitable value chains. The framework sets out four steps: (a) gendered value chain composition, (b) gendered value chain performance, (c) gendered value chain governance, and (d) gendered value chain upgrading (Fig. 1).

The first dimension, value chain composition, requires the creation of a gendered value chain map. This describes the value chain product(s) and its value, the different functions in the chain, and the roles different actors play in the chain (Kruijssen et al. 2021). Adding gender allows for a granular analysis of who does what within and across different nodes. It assesses which gender occupies high- and low-value products in the chain, and examines gendered roles and responsibilities for particular tasks, including ensuring all tasks are made visible—such as stocking ponds or packaging product at trader level.

The second dimension, value chain performance, assesses the outcomes of value chain participation. Traditionally, value chain performance is assessed in terms of economic indicators, such as the volumes being traded, the prices being paid, the returns in the value chain as expressed by margins, profits, wages and financial viability, value added, and employment created. In addition, it provides an understanding of social performance, which concerns performance on social indicators or the terms of engagement of different men and women actors in the chain. The third dimension, value chain governance, studies decision-making processes, and the power relations embedded in these, between value chain actors. It analyzes gendered power differentials in relation to the ability of women and men to take decisions and act on them at different value chain nodes and within the household. This dimension permits an understanding of who has the ability to decide which resources are to be used as inputs into a value chain, or allocated to other uses (Kruijssen et al. 2018). It entails an analysis of how power shapes the social relationships between value chain actors, and how these relations of power shape opportunities and constraints of farmers and other value chain actors’ meaningful participation and benefits from aquaculture.

The fourth dimension, value chain upgrading, is about improving capabilities, technologies, and institutions to improve the performance of specific actors, and the value chain overall. The focus is usually upon economic upgrading, but a gendered value chain approach also considers social upgrading to strengthen the social performance of the chain. This involves a more equitable distribution of benefits, for example, or improved working conditions for employees (Kruijssen et al. 2021).

An analysis of the aquaculture value chain from a gendered perspective can assist in identifying bottlenecks in the entire system, and specifically places where women’s participation is low, or where they fail to secure benefits commensurate with their participation. Such analysis provides the empirical data upon which strategic interventions for women’s inclusion and promotion of GEWE can be developed.

### Study areas

**Fig. 2:**
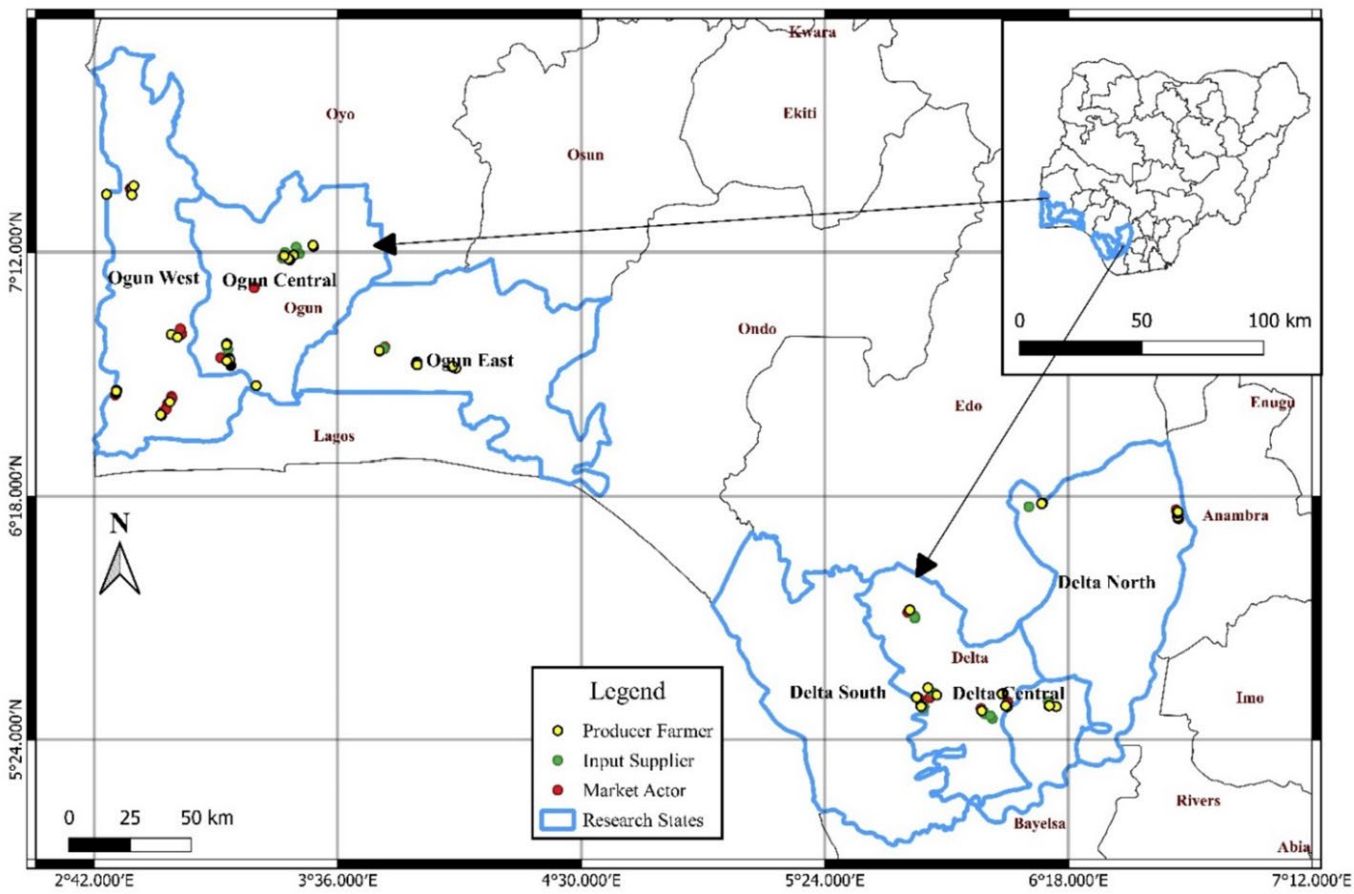
Selected research areas in Ogun and Delta States of Nigeria

The study was conducted in Ogun and Delta States in Nigeria. Ogun State lies within latitude 7°01′ and 7°18′ North and longitudes 2°45′ and 5°55′ East while Delta state lies within latitude 5°00′ and 6°30′ North and longitudes 5°00′ and 6°45′ East (Fig. 2). Ogun State was selected because of the region’s riverine topography, which is noted for its artisanal pursuits and significant catfish farming. Due to the presence of water and land resources that can support the establishment of freshwater fish species, Delta State was selected. The prevailing climatic and hydrographic conditions favor a small-scale fishery and agricultural economy within the two states.

### Research design and data collection

Data was collected in November and December 2021 as part of the Aquaculture: Increasing Income, Diversifying Diets, and Empowering Women in Bangladesh and Nigeria (IDEA) project. The study adopted a cross-sectional research design^2^ and a mixed method approach in data collection. Combining qualitative and quantitative approaches facilitated triangulation. The qualitative findings were used to help explain the trends identified in the quantitative analysis.

A multi-stage sampling technique was used to select respondents. The first stage involved the purposive selection of Ogun and Delta States. The sampling units were individual men and women participating in aquaculture value chains. In Ogun State, eight Local Government Areas (LGAs) from 20 LGAs were purposively selected: Ipokia, Imeko, Ewekoro, Ado odo, Odeda, Ijebu Ife, Ijebu ode, Yewa North. In Delta State, 8 LGAs from 25 were purposively selected: Oshimili South, Ika North-East, Uwvie, Sapele, Warri, Ugelli North, Isoko North, Ndokwa West. The selected LGAs exhibited the highest levels of aquaculture in each state (Olaoye and Odebiyi 2011; Khor et al. 2024).

Simple random sampling procedures were used to select individual households, traders, and input suppliers along aquaculture value chain in each LGA. Prior to commencing data collection, the focus group discussion (FGD) participants and key informants were briefed about the purpose of the research. They were then asked to sign consent forms allowing the research team to use the data they provided for research purposes. In addition, respondents were informed that the information they provide would be anonymized.

#### Quantitative research methods

To collect quantitative data, a structured questionnaire was developed for farmers, input suppliers, and traders. It was used to solicit information on the socio-economic status of the respondents, their participation in value chain development in relation to the node and their specific activities, their decision-making, relationships among different value chain actors, the distribution of benefits, and any challenges experienced in value chain upgrading.

For farmers, one member per household was interviewed. In men-headed households, either the head or the spouse was interviewed. Information was requested not only concerning themselves (for instance their own labor) but also concerning their spouse. In women-headed households, the head was interviewed with information on their spouse (for instance migrant husband) included where available. This strategy was employed in the knowledge that spouses may prefer different information on the same topic and that women’s work may be underestimated (Farnworth et al. 2023; Fox and Pimhidzai 2013).

Traders included fish wholesalers, processors, and retailers. Retailers vary widely. Some sell from formal trading locations/shops. Others are mobile retailers, with another category being restaurant and hotel owners. In total, we collected quantitative data from 410 farmers, 175 market actors, and 53 input suppliers.

#### Qualitative research methods

KII and FGDs were used to collect qualitative data using semi-structured interview guides (Table 1). In total, 11 FGDs were conducted with farmers: 3 mixed women and men FGDs, 4 women only FGDs, and 4 men only FGDs. In the FGDs men’s and women’s roles in input supply, production, marketing, ownership of assets and decision-making, and the distribution of benefits were discussed. With respect to KIIs, 20 input supplier KIIs, 14 fish processor KIIs, 16 fish retailers KIIs, 11 wholesaler KIIs, 30 consumer KIIs, and 25 KIIs from representatives from government offices, large feed manufacturing corporations, and NGOs were conducted. KII interview schedules explored value chain trends, roles and labor use, constraints, and opportunities in relation to aquaculture value chain development activities in the area and gendered participation in productive and non-productive activities.

**Table 1:** Data collection for the qualitative part of the research

		Ogun State			Delta State		
Value chain node	Data collection mode	Men	Women	Mixed	Men	Women	Total
Farmer	FGDs	33	3	3	1	1	11
Input supplier	KII	8	2		5	5	20
Fish processors	KII	1	3		4	5	14
Fish retailers	KII	5	5		3	3	16
Wholesalers	KII	1	4		1	5	11
Consumers	KII	2	14		5	9	30
Representatives from government offices, large feed manufacturing corporations, and NGOs	KII	8	2		12	3	25

### Data analysis

Through an iterative analytical process, where data was repeatedly reviewed, coded, and refined in relation to the analytic framework, the data was deductively organized and analyzed. Quantitative data was cleaned using Excel, and analysis conducted using STATA version 16.0 software. Data were analyzed and disaggregated based on gender along the aquaculture value chain and presented through descriptive statistics and cross-tabulations. Chi-square tests were used to determine the significance of asset ownership by men and women farmers at 95% confidence level. Qualitative data analysis was conducted using Nvivo 12. This enabled research themes to emerge and be further analyzed. Aquaculture value chain mapping was conducted by identifying and charting aquaculture value chains as explained by KIIs and through FGDs.

We further computed full-time employment equivalents (see Annex 1 for more information). Duncan’s dissimilarity index (Sakoda 1981) was calculated to examine how women’s participation would have to change in order for the distribution of women and men participants to be even across the aquaculture value chain (see Annex 2 for more information).

## Results

### Socio-demographic characteristics of the respondents

Table 2 shows the socio-economic characteristics of the respondents aggregated across Ogun and Delta States by gender including age and marital status. In Delta State, there was a notable gender imbalance among input suppliers, with males comprising 22.64% and females 16.98%. In contrast, producers in Delta State showed a more substantial disparity with 29.51% males and only 12.20% females. Market actors also reflected this trend, although to a lesser extent, with 20.57% male market actors and 26.86% female market actors. On the other hand, Ogun State presented an even starker contrast. While males dominated as input suppliers at 49.06%, females only made up 11.32%. This trend was also revealed among producers (40.49% male, 17.56% female) and market actors (29.71% male, 22.86% female). Such disparities reflect regional socio-economic dynamics influencing gender participation in aquaculture activities.

**Table 2:** Socio-demographic characteristics of the respondents per segment aggregated across Delta and Ogun States

	Input supplier (*n* = 53)		Producer (*n* = 410)		Market actor (*n* = 175)	
Characteristics	Male	Female	Male	Female	Male	Female
State						
Delta	22.64	16.98	29.51	12.20	20.57	26.86
Ogun	49.06	11.32	40.49	17.56	29.71	22.86
Age group						
18–25	1.89	1.89	3.90	2.44	2.29	1.71
26–35	15.09	5.66	12.44	7.07	13.14	12.57
36–45	26.41	11.32	24.88	12.93	20	25.14
46–55	15.09	3.77	16.83	5.12	9.14	5.71
> 55	13.21	5.66	11.95	2.20	5.71	4.57
Marital status						
Single	9.43	3.77	10.73	3.41	7.43	1.71’
Married	60.38	20.75	58.54	25.12	41.71	45.71
Separated	1.89	1.89	0.49	0.73	0	0.57
Widowed	0	1.89	0.24	0.49	1.14	1.71

Across age groups, there were varying degrees of gender disparity. In the 18–25 age bracket, the differences were relatively small but noticeable (1.89% vs. 1.89% for input suppliers, 3.90% vs. 2.44% for producers, and 2.29% vs. 1.71% for market actors). However, as age increases, so does the gender gap. For instance, in the 46–55 age group, males dominated significantly in all roles compared to females. The > 55 age group also shows a similar pattern, with males occupying a larger percentage of roles compared to females. This suggests potential challenges or barriers that women may face in entering or remaining in aquaculture-related professions as they age. Overall, our findings indicate that middle-aged individuals (aged 36–45 years) dominate the value chain, while the proportion of youths (18–35 years) is relatively small.

Marital status also plays a role in gender disparities within the aquaculture value chain. For instance, among married individuals, males hold a significantly higher percentage of roles across input suppliers (60.38% vs. 20.75%), producers (58.54% vs. 25.12%), and market actors (41.71% vs. 45.71%). This disparity could be influenced by traditional gender roles or access to resources and opportunities. Interestingly, the separated and widowed categories show relatively low participation overall, with males often outnumbering females. This could indicate potential challenges or limited opportunities for individuals in these marital status categories, regardless of gender.

### Gendered value chain composition

**Fig. 3:**
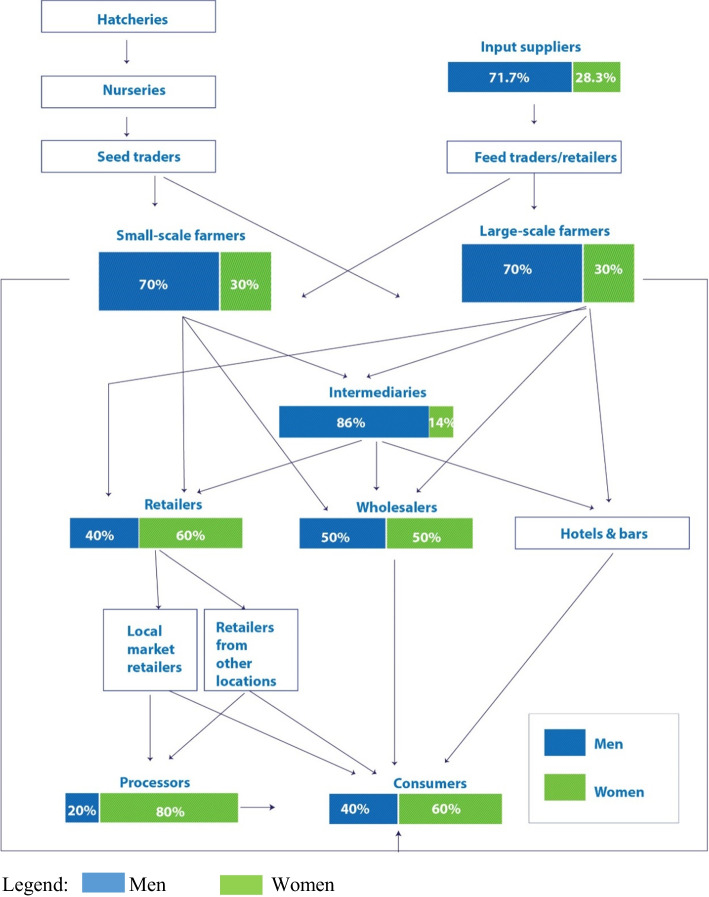
Men and women’s estimated rate of participation in the aquaculture value chain

Figure 3 depicts the aquaculture value chain. It details the points of access and nodes of activity for men and women. The study established that the major actors within this chain are input suppliers, fish farmers, and processors followed by retailers and consumers. Input suppliers include hatcheries, proprietors of nurseries, fish feed merchants, and other input suppliers (including seed traders). Fish farmers can be categorized into three: small-scale, intermediate, and large-scale. Fish are sold through three outlets: retailers (market stalls, other), wholesalers, and hotels and bars. Retailers, intermediaries, wholesalers, hotels, and bars make up the many categories of the retail sector.

Turning to gendered participation across the two states, men dominate input supply (71.3% men) and fish farming (70% men). Women form 80% of fish processors who in most cases add value to the fish products and sell to consumers and retailers alike, 60% of retailers, 14% of intermediaries who buy fish directly from the fishermen and sell to the wholesalers and retailers; and distributors who buy fish, store and sell to consumers, and 50% of wholesalers. Findings from the women FGD indicate that traders in each market come from different states, regions, and towns and are not necessarily from within the locality. The majority of the respondents in the consumption node of the value chain are women (60%) aggregated across Ogun and Delta States.

#### Gendered division of roles along the aquaculture value chain

**Input supply**: Fish input suppliers include fingerling producers and fish feed manufacturers and suppliers. According to key KIIs, most input suppliers primarily trade in fish feed, while about 10% also produce feed for both poultry and fish. The remaining 90% are involved in hatchery management and fingerling or fish seed production. Additionally, some informants mentioned offering training and extension services as private practitioners. Fish feeds are sold to small- and large-scale producers such as fish farmers and to small-scale feed traders/retailers. All input suppliers noted that their main customers were fish producers and were mostly men.

The quantitative data shows that the majority (79%) of input suppliers trade in local inputs, while 69% sell imported inputs. Approximately 56% of input suppliers trade in local and imported fish inputs. Most input suppliers obtain capital from savings, while others source from family and friends. The study found that payments were made using either cash or mobile money, and the payment terms do not differ by gender. Most input suppliers do not sell on credit. However, a few feed producers sell on credit to regular customers, normally at the start of fish production cycle. Women participants in one FGD stated that they mostly used imported feed in aquaculture production: “We use imported feed and local feed on occasion, though local feed is not recommended by us from our past experiences regarding productivity issues. However, when finances are tight and you still need to feed your fish, we try to use locally made sinking feed^3^ to keep fish in good condition” (FGD, women participants in Ogun State).

Both women and men informants involved in training and extension also provide loans or inputs on credit to fish producers and traders. Notably, these informants are not exclusively from the government but include private actors who offer fish inputs on credit, often with interest. Credit is generally given via informal arrangements; it accrues interest rate of between 0.1 and 10% and is usually payable in 2 months or less. Interest rates vary. For example, one male farm input supplier KII stated that the credit attracts 3% interest rate on each bag of feed. Another KII, woman supplier in Ogun State stated that the credit attracted 15% interest rate per production cycle. The input suppliers provide a range of inputs such as fingerlings and feeds depending on the fish farmers’ needs. In pointing out that the loan attracts an interest of 15%, this particular response from the women FGD response indicates lack of uniformity in the interest rates, with some loans attracting outrageous huge rates, and different repayment schedules. Such huge variances can affect uniform participation among the value chain actors.

**Gendered division of roles in fish production**: Regarding farming methods, most farmers practice fish monoculture. In monoculture systems, farmers produce either pure Clarias or hybrid species. Hybrid or *Heteroclarias* is a cross-breed of *Clarias gariepinus* (African catfish) (♀) x *Heterobranchus bidorsalis* (♂). Catfish and tilapia are the major polyculture species in both states. In Delta State, few farmers practice polyculture. Similarly, findings indicated that it is very rare for farmers to stock more than one fish species in Ogun State. However, tilapia from the wild often gets into the ponds, especially when ponds close to the river are left inactive for a few months.

Aquaculture activities in both states run throughout the year. While activities such as pond digging and maintenance/renovation are conducted once or rarely, others like management of predatory species, stocking, water management, weeding, feeding, fertilization, and harvesting are daily activities in the production cycle. In the study area, feeding is done twice a day for the first 4 months, after which the frequency is reduced to once a day. Gender dynamics in the fish production activities were predominant. Men dominate mainly in laborious activities such as pond digging, pond preparation, pond maintenance, predator species management, soil management, stocking, water management, weeding, feeding, fertilizing, and fish harvesting. It was noted that while male fish farmers focus long hours in fish farming, most female pond owners attempt to combine fish farming with domestic responsibilities by taking breaks from the pond to work on their household and care tasks. The female fish farmers go to the farm around 8:00 am and leave the pond location/site around 1:00 or 2:00 pm to cater for their children and do other household chores. Women farmers take up more responsibilities in fish farming when they do not have insufficient or no support from men, or when they are neglected by their husbands by totally withdrawing their input, both human resource and financial support. In the event that women solely own the ponds or have been neglected by their partners, they take up all activities, including the otherwise shared or male dominated, such as pond digging and its preparation, maintenance, feeding, harvesting, and sorting of fish. Sometimes, they must work longer/more hours depending on fish production activities.

Due to time constraints, women pond owners are more likely than men to hire laborers for fish-related activities, particularly for physically demanding tasks, which are typically assigned to male workers. They pay employees monthly and compensate daily laborers for tasks such as pond preparation and net dragging during harvest. This reliance on hired labor increases their fish production costs. Labor is commonly hired from within the village and sometimes from neighboring villages. In border communities such as the Ipokia Local Government Area in Ogun State, labor is sometimes hired from the neighboring country, Benin Republic.

**Men and women participation in fish marketing (on farm and beyond farm)**: Findings from the FGDs revealed that most male farmers sold their produce at the farm gate, while majority of the female farmers delivered to the markets. Some farmers advertise their businesses on social media to help traders and other consumers locate them for buying fish. Transactions are commonly conducted in cash, though some farmers practice sales on credit. Processors obtain fresh fish mainly from farmers, while dried fish was mainly obtained from retailers. There is a common perception by men that women prefer fish marketing as it is easier for them to build better relationships.

**Fig. 4:**
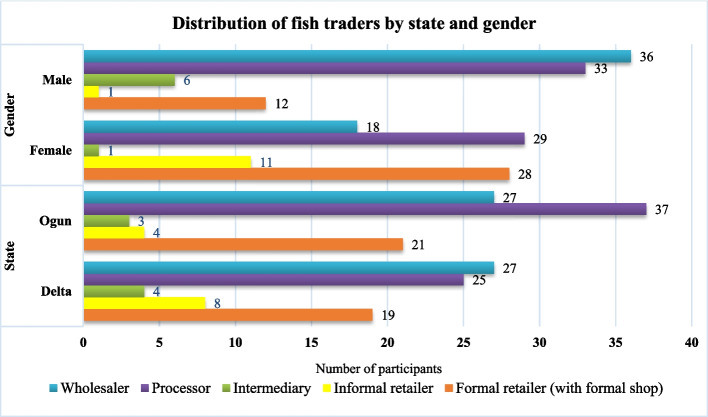
Distribution of fish traders by state and gender

Figure 4 shows the distribution of fish traders in terms of gender, state, and age group. Women dominate formal and informal retailing business and supersede men in numbers; however, men dominate the wholesaler category, and they are slightly more in numbers compared to women in the processing sector. The distribution of fish traders in Ogun and Delta States reveals notable differences in roles and gender participation. Ogun has a strong processing sector (*n* = 37) and a significant number of formal retailers with shops (*n* = 27), reflecting a well-developed formal market structure, with substantial female involvement in processing. Conversely, Delta also has a robust processing sector (*n* = 27) but shows more balance between genders in various roles, with a notable presence of intermediaries (*n* = 8) and informal retailers (*n* = 19), indicating a diverse market structure. These variations highlight the unique dynamics in each state, suggesting the need for tailored approaches to support and develop the aquaculture value chain effectively. Overall, these figures underscore the gender disparities across different roles in the fish trading value chain, with some roles being more male-dominated and others more female-inclusive.

**Fig. 5:**
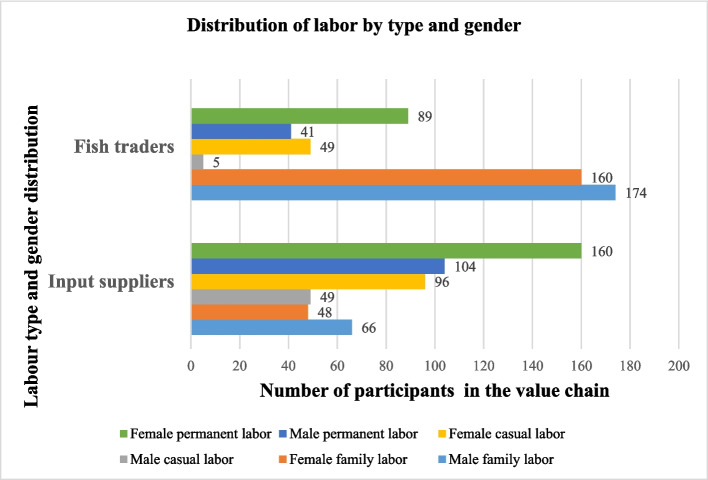
Distribution of labor and wage rates by type and gender

**Gender distribution of paid and unpaid labor**: Fig. 5 illustrates the gender distribution of waged and unpaid family labor in the input supply and fish trader segments. In total, 199 and 394 men and women have waged employment (permanent and casual labors) respectively in these nodes of the value chain in the study areas.

Most of input suppliers and fish farmers do not involve their family members (*n* = 48 for female family labor and *n* = 66 for male family labor), especially children in their businesses. Those who do often allocate light tasks such as pond cleaning (involving removal of algae, ensuring water is clean, picking out plant debris that might gotten into the pond) and feeding and grading of fish. Some wholesalers and processors also get support for their business from their family members. Findings indicated that male family labor (*n* = 240) was more involved in the input and fish trading segments compared to female labor (*n* = 208).

The number of hired laborers depends on the business scale needs. For instance, women tend to be hired for marketing roles while men tend to be hired for loading and unloading, pond digging, and other labor-intensive tasks. Women comprise 26% and 34% of casual and permanent labor respectively. In terms of the wage rate and based on the computations of Full Time Equivalents (FTEs) in the input supply segment, women and men casual laborers earn a daily wage rate of (Nigerian Naira) NGN 667 (United States dollar (USD) 1.45)^4^ and NGN 876 (USD 1.91) respectively, while women and men in permanent labor supply earned NGN 25,000 (USD 54.41) and NGN 26,000 (USD 56.59) as a monthly salary, respectively.

In fish trading, male and female casual laborers receive an average monthly of NGN 1252 (USD 2.73) and NGN 1055 (USD 2.30) while in permanent labor supply, male and female workers received an average monthly salary of NGN 34,770 (USD 75.68) and NGN 30,776 (USD 66.98), respectively.

**Full Time Employment Equivalent (FTE)**: Employment is dominated by men (Table 3). The fish input supply and market trading of fish provided an overall total of NGN 4,340,793 FTE per annum, in the form of family, casual, and permanent labor, out of which 60.94% (NGN 2,645,713 FTE per annum) was provided by men. The input supply and fish trading had 30.14% (4409 FTE per annum) and 39.08% of the total paid FTE in per annum per segment, provided by women respectively in each value chain node. The calculations of FTEs are as indicated in Annex 1.

**Table 3:** Gender distribution of per annum FTEs in aquaculture value chain

Type of labor	Input suppliers			Fish traders			Overall
	Men	Women	Sum total	Men	Women	Sum total	
Casual	1549	260	1809	779,340	551,720	1,331,060	1,332,869
Permanent	5327	2132	7459	1,830,920	1,114,360	2,945,280	2,952,739
Household	3343	2017	5360	25,034	24,791	49,825	55,185
Sum total	10,419	4409	14, 628	2,635,294	1,690,871	4,326,165	4,340,793

**Gender roles and Duncan’s dissimilarity index**: Findings indicate that men dominate all the three main stages of the value chain in all the three forms of labor (casual, permanent, and family) including the input supply stage (71.70%), production stage (70%), and marketing stage (50.29%), as in Table 4. The percentage of dissimilarity between the roles of men and women in aquaculture value chain is 17.35%, implying that women have an absolute dominance of 17.35% across the entire chain. The individual levels of dissimilarity among the aquaculture value chain nodes were computed as follows: for input suppliers, the level of dissimilarity was 1.25% and men dominated the input supply in the aquaculture value chain. The level of dissimilarity for producer farmer and market actors was 7.40% and 8.70% respectively with men still dominating these nodes of the aquaculture value chain. The gendered value chain participation implies that men participate much in the primary production; in this case, men dominate activities of fish production value chain from pond construction time fingerlings to harvesting of fish. On the other hand, women form the majority in the downstream activities such as processing and marketing the fish products.

**Table 4:** Gender roles and Duncan’s dissimilarity index

Actors	Male		Female		Total (frequency)	Absolute difference	Duncan’s index of dissimilarity
	Frequency	%	Frequency	%			
Input supplier	38	71.70	15	28.30	53	0.025	1.25
Producer farmer	287	70	123	30	410	0.148	7.40
Market actors	88	50.29	87	49.71	175	0.174	8.70
Total	413		225		638	0.347	17.35

#### Gender norms and their effects on types of work

In the input supply and fish processing segments, women do not participate in lifting/carrying of heavy loads, while in the fish farming segment, they do not take part in pond digging. These tasks are performed by men because these tasks are normatively considered hazardous to women’s health. For instance, women do not participate in some of production nodes such as pond preparation as they are not considered strong enough to undertake tasks demanding physical rigor.

Regarding the hiring of casual and permanent labor, KIIs reported that some tasks are delegated to an “appropriate” gender through gendered hiring criteria. For instance, some fish farmers hire male labor for pond clearing, daily routine pond checks, and water pump checks. Women laborers are hired for fish feeding (FGD respondents).

A gender norm that women are responsible for household and care work is widely prevalent. Very few men perform these roles. As a consequence, women reported being burdened with chores and having insufficient time for income-generating activities. The close association of women with reproductive norms anchors and legitimizes the gendered division of labor. For instance, input suppliers noted that hatchery businesses are time-consuming and need full focus on the work. This makes it difficult for women to run successful hatchery businesses because they cannot invest the time required. A few exceptions were noted. Women of low economic status perform hard labor which is normatively ascribed to men (i.e., lifting/carrying feeds and other materials). Educated women and those with family support take on roles similar to men, including bookkeeping, particularly in managing and leading businesses. Two input suppliers explained that the differences in the roles played by women and men emanate from men having more capital, and hence the ability to invest and perform various activities that women may not be able to. This relates to the norm that men own productive assets.

Many women negotiate around this norm by working as processors, as the capital required is a lot less. In very large feed manufacturing corporations, women work in administrative, bookkeeping, and marketing positions but not as entrepreneurs. In Ogun State, a case was observed where a woman works as feed mill operator and supervisor. The woman hires daily waged male employees to unload and carry feed ingredients. Men’s labor was also used to support her in measuring feed ingredients during the milling process.

Nevertheless, women and men reported that they support and encourage each other in gendered tasks. Processors perceived that the gender division of labor does not influence their business. Although men and women have different roles and responsibilities, they are often said to be complementary to one another. Some processors made decisions in collaboration with their spouses, while others made them on their own.

#### Gender disaggregated social and economic performance of the aquaculture value chain

**Ownership of resources for fish production, marketing, and sales**: Ownership of, and access to, resources is essential for value chain participation. Inter-household asset ownership in the aquaculture value chain, gender differences, and disparity often manifest in unequal access and control over these shared assets. Addressing these disparities is essential to ensure equitable resource distribution, empower women, and enhance overall community resilience and economic development in aquaculture. Table 5 shows the percentage distribution of asset ownership by gender of input supplier and market actor. Smartphone ownership was significantly different between women and men input suppliers (*p* = 0.000) while manual weighing scale and digital weighing scale ownership varied significantly among market actors (*p* = 0.005 and *p* = 0.001 respectively).

**Table 5:** Asset ownership by percentage gender of input supplier (*n* = 53) and market actor (*n* = 175) in percentages

Asset	Value chain node	Male	Female	Total	*x*^2^ value	*p* value
Land	Input supplier	26.4	9.4	35.8	0.043	0.836
	Market actor	24.6	20.6	45.2	0.022	0.882
Car	Input supplier	39.6	13.2	52.8	0.226	0.634
	Market actor	24.6	15.4	40	2.826	0.093
Motorbike	Input supplier	20.8	5.7	26.5	0.020	0.888
	Market actor	17.1	15.4	32.5	0.191	0.662
Smartphone	Input supplier	58.5	26.4	84.9	1.159	0.282
	Market actor	42.3	21.1	63.4	32.579	0.000*
Deep freezer	Input supplier	15.1	7.5	22.6	0.194	0.660
	Market actor	13.7	13.1	26.8	0.016	0.901
Manual scale	Input supplier	39.6	17	56.6	0.098	0.754
	Market actor	34.3	23.4	57.7	7.947	0.005*
Refrigerator	Input supplier	11.3	3.8	15.1	0.051	0.822
	Market actor	6.3	1.7	8	4.870	0.027*
Digital scale	Input supplier	45.3	17	62.3	0.046	0.831
	Market actor	13.1	3.4	16.5	11.714	0.001*

* indicates statistical significance at *p* < 0.05 level of significance

**Fig. 6:**
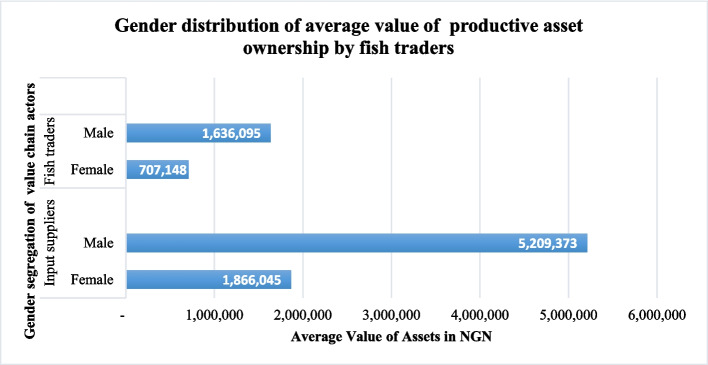
Average value of productive assets in NGN owned by input suppliers and fish traders categorized by gender

Regarding the value of asset ownership over the production and marketing period in aquaculture value chain, gender differences occur in both the input supply and fish trading segments. As indicated in Fig. 6, the average value of productive assets owned by each women input supplier was NGN 1,866,045 (USD 4062.37), while men owned an average of NGN 5,209,373 (USD 1133.91). On the other hand, the value of productive assets owned by women fish traders was NGN 707,148 (USD 1539.12) while male traders had higher value of assets valued at NGN 1,636,095 (USD 3561).

Social capital (Table 6) was assessed among the input suppliers with respect to their having fish group/association membership and access to credit (because in the absence of formal sources of finance, access to credit depends on activating social relationships). The findings show that 32.1% had access to credit while 69.8% were members of groups/associations aggregated across the two states. Chi-square test revealed a significant association between gender and group membership (*p* = 0.029) among input suppliers. Among the producers, 27.6% had access to credit, while 47.3% were members of fish farming groups/associations. Chi-square test revealed a significant association between gender and access to credit (*p* = 0.041) among the farmers. The study found that women and men KII who provide training and extension provided loans or inputs on credit to fish producers and traders. The credit is generally given via informal arrangements; it accrues a small interest and is usually payable in 2 months or less. The interest charged for the loans ranged from 0 to 15%. A small portion of the loan recipients of credit from the women lenders are women, while the man lender’s loan recipients are all men because there are reportedly lower numbers of women in the overall value chain.

**Table 6:** Access to credit and group membership by gender of the input supplier (*n* = 53), farmer (*n* = 410), and market actor (*n* = 175) in percentages

	Value chain node	Male	Female	Total	*x*^2^ value	*p* value
Access to credit	Input supplier	18.9	13.2	32.1	0.23	0.880
	Farmer	20.5	7.1	27.6	4.164	0.041*
	Market actor	19.4	18.3	37.7	0.948	0.330
Group membership	Input supplier	54.7	15.1	69.8	4.757	0.029*
	Farmer	32.2	15.1	47.3	1.660	0.198
	Market actor	34.3	28.6	62.9	1.119	0.274

* indicate statistical significance at *p* < 0.05

**Fig. 7:**
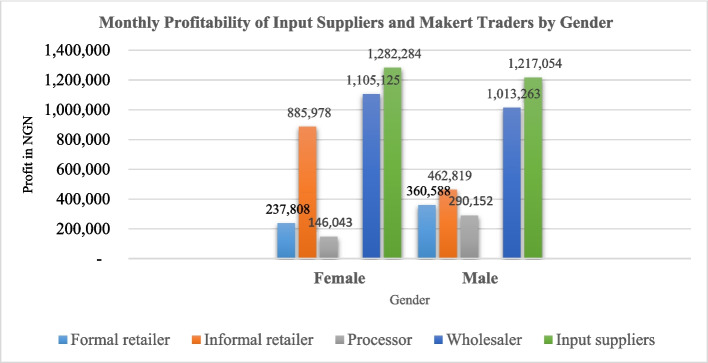
Monthly profitability of input suppliers and market traders by gender

**Distribution of benefits**: On average, women traders in the fish trading segment realize the highest monthly net profits NGN 593,738.5 (USD 1292.29) compared to their male counterparts NGN 531,705.5 (USD 1157.27). On the other hand, men input suppliers realize higher monthly net profits NGN 1,282,284 (USD 2790.92) than women input suppliers NGN 1,217,054 (USD 2648.95) as illustrated in Fig. 7.

**Gendered Distribution of Benefits**: The distribution of benefits from aquaculture production and marketing varies between men and women, partly reflecting their labor input, but also the use of output for home consumption or for sale, and the dominance of men as household heads. Men and women fish farmers do not have equal access to or control over the income gained from aquaculture production and marketing. In one FGD, a female fish farmer stated that she has control over all the income generated from aquaculture production and would solely make decision about how to spend it after consultations from the husband and household.

“It’s my business, when it comes to money, I make the decision on spending the income. I am always open to advice and guidance from my husband and household.” (female FGD participant, fish farmer, Ogun State).

### Gendered value chain governance

**Decision-making in aquaculture value chain input supply, production, and marketing**: Most respondent family members are involved in the aquaculture business as farmers. Therefore, the decisions on the fish produced to be used for either household consumption, for sale, or given as a gift is reported to depend on many factors, including the number of people within the household, and overall household income.

Decision-making ability varies according to the actor’s marital status, with married women expected to consult their spouses before deciding as compared to unmarried women (widowed, single, and divorced women). Yet a men’s FGD revealed that in some cases, women who own businesses across the value chain node decide in consultation with their children rather than with their spouses. In a women’s FGD, informants reported that they make decisions together with their family, which includes their spouse and their adult children. In one men’s FGD, men explained that women were free to make their own decisions regarding production activities. As one man explained:

“Women are free to make decisions in the home. No gender division of labour, everyone can do same domestic and care work regarding production activities.” (FGD, male participant in Delta State).

When making decisions, fish farmers who consult their family members tend to discuss and come into consensus on what is needed and what action needs to be taken. Oftentimes, gender is a less important criterion than knowledge. Those recognized to have more knowledge make the final decision.

Women’s mobility on where they work (including to sell the fish they produce) depends on their marital status. Married women rely on the permission given by their husbands when supported financially by their husbands, whereas unmarried women make their own decision with regard to the value chain activities. As one woman explained, joint decision-making sometimes occurs:

“In cases where the man supported you financially in fish production activities, decision making will now come from you both.” (female FGD participant, fish farmer, Delta State).

Other factors that influence decision-making within the households and businesses include profitability and risks of losing income.

### Gendered value chain upgrading

**Constraints in value chain upgrading**: Women farmers reported several challenges towards value chains upgrading including bad water quality, flooding, high cost of feed and transportation, lack of funds, and the theft of fish from their ponds which stem from smaller, less secure operations and limited access to financial resource. Men highlighted that water shortages, poor quality and shortages of fish seed, and shortages of fish feed were their main challenges often due to the larger scale of their operations and greater demand for resources. A woman female KII in Ogun, stated:

“The business has not been easy because of the cost of feed and the market is not too good because of poor patronage from the consumers due to insufficient money. The same species have been marketed for the past three years. The size of the fish has also reduced. However, the price per kg of fish has also increased over time. The return on investment in the business has also been affected adversely in the past three years for example one million naira put into the business brings an output worth eight hundred thousand naira. That is a loss of two hundred thousand naira.” (female KII market trader in Ogun State).

Challenges unique to each state were also noted. In Delta State, poor quality of fish seed, warm weather which is a barrier for carp fish aquaculture and water shortage were additional challenges which were mentioned. In Ogun State, farmers highlighted floods, poor security, and environmental pollution as constraints to fish farming.

Farm assistants who work as both permanent and casual laborers were perceived as a blocker because they have the tendency to steal and sell the fish from the fish farmer’s pond. One of the women informants from an FGD stated that she once hired a farm assistant who stole and sold fish from her pond until only around 20% was left. Farm assistants were also perceived to provide information to thieves, allowing the latter to steal easily.

**Aspirations for value chain upgrading**: The majority of young women and men (18–35 years) aspire to upgrade within the production node of the value chain. This includes having a larger number of ponds and a larger area of land to develop for their aquaculture business within the next 5–10 years. Apart from the size of the business, they also aim to expand to other states and countries. Some young women and men aspire to upgrade to other nodes along the fish value chain and into other agricultural commodity chains as well.

In Delta State, a woman wholesaler KII stressed the need for government intervention as a way of upgrading the aquaculture value.

“Many people want to do the fish business, but money is the problem and the ones doing the business already are afraid now because of the high cost of fish. If the government will not come to assist the people through offering credit facilities, many may run away from the business very soon” (female wholesale KII in Delta State).

## Discussion

This paper fills the critical but largely lacking gendered information in the aquaculture value chain as noted by Adam and Njogu (2023). Data was obtained through mapping gender roles, levels of engagement, and benefits along the aquaculture value chain, and was analyzed through a gendered aquaculture value chain analysis framework (Kruijssen et al. 2021).

**Gendered value chain composition**: Overall, the aquaculture value chain in Nigeria is men dominated. This is partly due to its capital-intensive nature and the technologies associated with its development (Kumar et al. 2018). As primary breadwinners, men have better access to resources, allowing them to manage and invest more in the sector. Due to the tremendous investment and adoption of new technologies relating to its development, aquaculture tends to be a male-dominated sector (Kumar et al. 2018). The study found a significant discrepancy in favor of men in the input supply and production nodes of the aquaculture value chain. However, the proportion of women and men in the marketing node is comparable. This supports the findings in other studies, which report that women are mostly active in the marketing node in Nigeria (Adeoye 2020). In addition, women encounter different barriers to accessing the production elements than men because men are typically household heads and make all the decisions (Farm Africa 2016). Our findings also indicate that middle-aged individuals (aged 36–45 years) dominate the value chain of all the three major nodes. These findings are consistent with those of Omeje et al. (2021) and Idiku et al. (2022) in Nigeria. Overall, the results support previous findings which have reported male dominance of the most profitable nodes of the aquaculture value chain in Nigeria (Adam et al. 2021; Omeje et al. 2021). This study also corroborates findings conducted in other African countries such as those by Githukia et al. (2020), Ndanga et al. (2013), and Olufayo (2012) which reported that women were engaged in fish transport, processing, and marketing as opposed to men who mainly produced fish. The invisible contribution of women is important, especially when examining the effect of gender relations on aquaculture production since it is impossible to imagine the aquaculture sector without women especially on the terminal end of the value chain (Harrison 2000).

**Gendered participation and division of roles along the aquaculture value chain**: In the aquaculture value chain, men and women clearly have different duties, roles, and responsibilities. In the value chain and beyond, encompassing paid and unpaid labor, the gender division of labor considers the various productive responsibilities, roles, and positions held by men and women. The result of Duncan’s index of dissimilarity of 17.35% indicates that the aquaculture value chains are gender segregated implying that men and women cluster at different levels of the value chain.

The gender distribution of annual FTEs in the aquaculture value chain indicates that male workers predominated in paid employment in the input suppliers’ and fish wholesalers’ segments. According to our findings, men are involved in more labor-intensive tasks including pond digging in the value chain while women are involved in less labor-intensive tasks such as pond cleaning and feeding, which is consistent with previous findings (Clucas and Ward 1996; Nwabueze 2010; Omitoyin et al. 2011). Men dominate both casual and permanent labor.

Most of the work done by women is unpaid, undervalued, and seen as an extension of household duties. The present study reported that it was a common norm along the value chain for women to be incapable for activities that demand physical labor and strength. In the input supply and fish processing segments, women did not participate in lifting/carrying of heavy loads, while in the fish farming segment they did not take part in pond digging. The results collaborate with the study of Pavo and Digal (2016) which also reported that tasks of women in the aquaculture value chain include slicing, skinning, and packing which usually do not require great physical strength. Quaye et al. (2016) note that the capacity of women to shape and make better-informed life decisions, as well as women’s empowerment, is crucial to changing gender roles in society.

**Gender disaggregated social and economic performance of the aquaculture value chain**: The findings reveal a gender difference in ownership of productive assets. This could be attributed to cultural, social, patriarchal, and religious norms that prevent women from participating equitably in the aquaculture value chain in Nigeria (Sasa et al. 2022). As deduced from the qualitative results, men are expected to lead and provide for their families, while women are expected to maintain the homes and support their children and husbands. Since men are seen as the household heads and expected to bring income, they may therefore take ownership of productive assets such as land. The findings are similar to those of Chete (2019), who posited that women possessed smaller pieces of land than men due to a lack of statutory land rights and patriarchal land systems. Gender difference in asset ownership was also noted in the input supply and fish trading segments. The average value of assets owned by each female input supplier and fish trader was lower when compared to that of men.

Omitoyin et al. (2011) report that most aquaculturists belong to cooperative societies, though the percentage of women members is greater than that of men. This may be because women have been identified as encountering constraints in accessing productive inputs. They may believe participating in such cooperative societies will enhance their access to productive resources. Ndanga et al. (2013) and Githukia et al. (2020) also echoed the same sentiments noting that men have easier access to productive resources than women. Ajani (2009) also echoed these studies by reporting that patriarchal arrangements in Nigeria favor men at the expense of women by allocating them more productive land. The findings align with that of Omitoyin et al. (2011), who attributed this to the cultural norms that have relegated females to the background which do not allow them to have access to productive resources like their male counterparts. Studies demonstrate a substantial gap in gender access to production resources (Olufayo 2012). Gender imbalances exist along aquaculture value chains and women do not have equal access to equal pay, capital, or voice as men (Jolly et al. 2023).

**Benefits**: The study confirms the existence of the gender gap in distribution of benefits in the aquaculture value chain. Gendered distribution of benefits in value chains relates to the economic returns from activities participated in by males and females (e.g., income from sales, wages) (Kruijssen et al. 2018). Aquaculture has the potential to contribute significant income to the actors involved. Our findings indicated that male input suppliers realized higher profits than the female input suppliers. The trend was the same in the fish trading segment.

The gender gap in earnings between men and women working in fish farming is also clearly seen in the distribution of benefits (Omananyi 2021). Women make much less money from fish farming than males, which is more evidence of entrenched gender inequality (Sasa et al. 2022). The findings are similar to those of Omeje et al. (2021) who found that men received realized a higher net income from aquaculture than women possibly due to the higher levels of investment in aquaculture by the men. The importance of women’s active involvement in enhancing household financial success and food and nutrition security cannot be stressed enough (Genschick et al. 2018). Previous studies indicate that aquaculture growth does not benefit both men and women as it should because of the unequal distribution of earnings and access to resources for farming between men and women (Adam et al. 2021). Aquaculture benefits are not equally distributed among women and men of varying ages and social strata who work in and depend on it because of disparities in endowments and barriers connected with access to production resources (Ndanga et al. 2013). Women work in aquaculture in a variety of ways, making important contributions to the general welfare of households, but they frequently receive relatively little in return due to pervasive gender inequities in the sociocultural and economic arenas (Githukia et al. 2020; Olufayo 2012). Women’s contributions must be fully appreciated and acknowledged to achieve economic prosperity and food security, which calls for coordinated efforts and assistance at the family, community, and national levels.

**Membership of groups**: Our findings further revealed that membership in groups or associations among Nigeria's aquaculture value chain actors is relatively high. The findings, however, reveal a significant association between membership in groups or associations and gender among the input suppliers, implying that gender influences the input suppliers’ choice or ability to belong to a group or association.

Social capital enables individuals to collaborate to accomplish a shared objective or purpose (Sandefur & Laumann 1998). Interpersonal ties, common standards, principles, and trust enable a community or organization to work as one (Welzel et al. 2005). Social capital is vital since it gives individuals a sense of belonging and shared identity. Social capital studies emphasize membership in voluntary associations (Welzel et al. 2005; Ehsan et al. 2019). Membership in associations is the primary indication of societal linkages in most comparative studies of social capital (Lindström, 2020; Welzel et al. 2005; Zidrou et al. 2021).

As an avenue to gender-transformative approaches (GTA), participation of women in groups or associations provides them with opportunities to meet and establish relationships with other women, which are avenues for informal sharing of experiences and ideas-activities that women value as opportunities for learning (Manlosa et al. 2019). Thus, the need to address inequities at various levels in order to create synergies is recognized by GTA (Fischer et al. 2020). GTA are a way to incorporate gender into development that aims to change the social context and create an environment that is supportive of gender equality and women’s empowerment. They achieve this by assisting men and women in critically examining gender norms and disparities, acting to improve norms that support equality, and challenging and changing the underlying social structures, policies, and norms that perpetuate gender disparities (Galiè & Kantor 2016; McDougall et al. 2021). GTA are superior to gender-accommodative approaches as the latter focuses on strategies to ensure women’s participation, while the former attempts to develop critical consciousness and tackle gender norms (Cole et al. 2020).

**Gendered value chain governance**: The participation of women in decision-making is expected to act as an empowerment that would enhance their involvement in the aquaculture value chain. The study findings indicate that both men and women take part in making decisions, but decision-making is dependent on marital status, household size, household income, and an individual’s social status, where those with more knowledge or income make the final decisions. This implies some gender difference in decision-making between males and females and between people of different social statuses in the Nigerian aquaculture value chain, where some individuals’ opinions are not considered despite being actively involved in activities along the value chain. This was also in agreement with the study of Githukia et al. (2020), which reported that females and males have social norms, interaction, and mobility challenges that mainly affect women and are associated with domestic responsibilities. As such, women’s capacity to pursue a broader range of livelihood activities and to attend training is limited by physical mobility restraints. Similarly, Morgan et al. (2017) noted that gender norms shape men’s and women’s differential decision-making power, with a knock-on effect on aquaculture productivity. The same sentiments were echoed by Lawless et al. (2019), who noted serious gender norms restricting women from leaving the households since their husbands could not undertake domestic duties because it was against customary expectations. Also, this was a way of challenging existing power relations and a high form of disrespect for their husbands (Boudet 2013) and could increase relationship tension. Family responsibilities reduce women’s availability in meetings and limit their participation as they carry out other domestic activities. Findings point out the importance of increasing awareness of women’s rights and how to change decision-making. Kusakabe (2003) pointed out that the resources and knowledge that women have relative to their husbands are significant determinants of women’s decision-making power in household aquaculture activities.

**Constraints in upgrading the aquaculture value chain**: Both the groups of women and men revealed different challenges in upgrading the value chain, including the high cost of feed and transportation, lack of funds, the theft of fish from their ponds, water shortage and poor water quality and shortages of fish seed, and shortages of fish*.* These findings align with that of Olaoye et al. (2013), who found that fish feed is the main expense, ranging from 60 to 70% of total expenses in aquaculture. Similarly, in a study of fish farmers in the Kainji Lake Basin region, Omeje et al. (2021) found that men (59%) and youth (63%) primarily used their savings to finance their aquaculture businesses, while the majority of women (70%) relied on help from a relative or friend. This finding suggests that women heavily rely on friendship and family ties, in contrast to the slight autonomy shown by youth and men.

**Opportunities to upgrade in the value chain**: Group membership can empower women and favorably impact their income and involvement in fish farming (Tikadar et al. 2022). The Eriwe Fish Village Project in Ogun State illustrates how farmer groups and cooperatives boost earnings by giving farmers greater access to improved production techniques, cooperative sales for higher prices, and loans (Folorunso et al. 2021). The likelihood of trading, making money, and having access to fish supplies, capital, and credit increases with group membership (Ankrah et al., 2021). Additionally, loan availability is promoted via financial literacy training and the formalization of groups to facilitate group borrowing (Adam and Njogu 2023). Possibilities exist for diversification in the value chain’s trade sector. Women are mostly employed in the limited postharvest processes for fresh, frozen, or smoked fish (Subasinghe et al. 2021). Thanks to the growth of small-scale fish value addition and processing, women would have greater economic opportunities, which would also increase the variety of postharvest fish value chain segments in which they participate. Making fish oil and crackers, which have a large market need and do not require consistent energy or large expenditures, are two examples of such prospects (BoP Inc. and WorldFish, 2021).

## Conclusion

The study provides several new insights. First, it identifies a significant gender disparity in asset ownership and wage rates along the aquaculture value chain. Men have greater access to productive resources and earn higher profits than women. Second, the study finds that women’s participation in decision-making is relatively high. This is due to their engagement in various aquaculture activities, challenging the assumption that men dominate decision-making. Third, informal credit arrangements play a crucial role in women’s access to financial resources. However, these arrangements often come with varying and sometimes exorbitant interest rates. Lastly, while women dominate fish processing and retailing, they are underrepresented in high-value nodes such as input supply and production. This reinforces structural inequalities in the sector. These findings highlight the need for targeted interventions to improve gender equity in resource access, financial inclusion, and value chain participation. Building on the study’s findings, it is imperative that gender differences in access to productive resources in aquaculture value chain call for empowerment by NGOs, governments, and other development agencies to effectively target women in their interventions for aquaculture development in Nigeria. For example, it would be imperative to provide financial support or grants and create collaborative platform/network of input suppliers while for the retailers, processors, and wholesalers strengthening the cooperatives/associations and unions would help to regulate prices. However, targeting women, without addressing deeper structural barriers, will create a viscous cycle of women’s disempowerment in economics and technical and social status, calling for the need for applying GTA in project or program implementations. GTA works on the deep structural barriers to engage with, reduce, or overcome structural constraints from household to national and even global levels. The structural barriers are the formal (policies), semi-formal (systems), and informal (norms). If they exist, there is a need to create policies and/or implement enabling environments required for women to equitably benefit from resources. There is a need to create data systems that can count women’s contributions, and extension services that consider the needs of women and recognize them as “real” fish farmers and/or big players in the fish value chain system. Furthermore, there is a need to pay attention and address the burden of unpaid household work on women and provide them the needed support from their family, community, and government, which will pave the way for their ability to live healthy and productive lives.

## Limitations of the study

This study was restricted to Ogun and Delta Nigeria and considered the aquaculture value chain. Therefore, extending the research to other states and countries to provide more generalizable conclusions is an excellent opportunity for future research. Secondly, the survey did not use empirical data from a dual-headed household survey to establish intra-household gender dynamics; hence, the gender dynamics comparison is on interhousehold basis. This study thus recommends future studies to consider intra-household analysis. Further study is needed to understand how gender-based violence, including the denial of resources, opportunities, and services, as well as social, cultural, and behavioral restrictions, affects women’s ability to participate in aquaculture value chains and generation of benefits.

### Annex 1. Computation of Full Time Employment Equivalents

The Full Time Equivalents (FTEs) were generated to assess gendered employment in the value chain as the time individuals in labor force spend on aquaculture value chain as an economic activity. FTEs were calculated from the primary data as the amount of time an individual works in an activity, relative to a standard benchmark of 40 h per week (FTE = 1). The study output generated data on the number of people employed and on the nature of that employment, whether full-time, part-time, or season, whether employees are men or women, and over or under the age of 30 years. Working full time is assumed to be 12 months per year, 21 days per month, and 8 h per day. An individual who is not in the workforce over the past course of the year would have an FTE of 0.5 for that activity. We then aggregated the estimated values for each value chain segment to derive the employment generation for the entire value chain. The FTEs calculation in this study largely borrowed from the works of Dolislager et al. (2021) and Nasr-Allah et al. (2020).

### Annex 2. Description of the distribution and major roles of men and women at each stage of the value chain

Duncan’s dissimilarity index was calculated to examine how women’s participation would have to change in order for the distribution of male and female participants to be even across the aquaculture value chain. The index provides a metric of how integrated their participation is across the aquaculture value chain relative to men (Sakoda 1981). The index has a range of 0–100, where a value of 0 means a sector is not gender segregated and a value of 100 means otherwise. The index also provides the percent of either women or men that would have to switch to other stages in the value chain in order for the distribution of men and women to be equal across the value chain. Previous studies (Donkoh et al. 2020; Mensah-Bonsu et al. 2019) have used Duncan’s index of dissimilarity to estimate the distribution of roles of men and women in agricultural value chains and Szymkowiak (2020) in fisheries. Duncan’s index of dissimilarity was therefore used to measure gender segmentation for the entire aquaculture value chain as in Eq. (1).

1 $$D=100 \times 0.5 \sum_{i=1}^{N}({f}_{i}-{m}_{i})$$

In the context of this study, Eq. 1 is expanded as in Eq. 2:

2 $$D=100\times 0.5{\sum }_{i=1}^{N}\left[\left(\frac{{f}_{input }}{F}-\frac{{m}_{input}}{M}\right)+\left(\frac{{f}_{farmer}}{F}-\text{i}=\frac{{m}_{farmer}}{M}\right)+\left(\frac{{f}_{market}}{F}-\frac{{m}_{market}}{M}\right)\right]$$

## Data Availability

Data is available upon request.

## Footnotes

1 Value chain: Actors and activities involved in the production, processing, distribution, and consumption of food.

2 A type of observational study that analyzes data from a population, or a representative subset, at a specific point in time (https://en.wikipedia.org/wiki/Cross-sectional_study).

3 Sinking feed, also referred to as hard pellets, is a locally produced fish feed that offers economic benefits for fish farmers. These pellets maintain water stability for approximately an hour and gradually sink to the bottom of the pond if left uneaten.

4 The conversion rate was approximately 459.44 NGN per 1 USD.

## References

[CR1] Adam R, Byrd K, Siriwardena S, Subasinghe R, McDougall C (2021) Gender in Nigeria’s aquaculture and small-scale fisheries value chains. Penang, Malaysia: WorldFish, Poster. 10.5897/SRE12

[CR2] Adam R, Njogu L (2023) A review of gender inequality and women’s empowerment in aquaculture using the reach-benefit-empower-transform framework approach: a case study of Nigeria. Frontiers Aquaculture 1:2. 10.3389/faquc.2022.1052097

[CR3] Adeleke B, Robertson-Andersson D, Moodley G, Taylor S (2020) Aquaculture in Africa: a comparative review of Egypt, Nigeria, and Uganda vis-a-vis South Africa. Reviews in Fisheries Science & Aquaculture 29(2):167–197. 10.1080/23308249.2020.1795615

[CR4] Adeoye AS (2020) Assessment of gender roles in fish farming activities among rural farmers in Afijio Local Government Area of Oyo State, Nigeria. Nigeria Agricultural J 51(2):406–412. http://www.ajol.info/index.php/naj. Accessed 20 Mar 2023

[CR5] Ajani, OIY (2009) Gender dimensions of agriculture, poverty, nutrition and food security in Nigeria. NSSP Working Paper 5. Abuja, Nigeria: International Food Policy Research Institute (IFPRI). https://hdl.handle.net/10568/162046

[CR6] AnkrahTwumasi M, Jiang Y, Addai B, Ding Z, Chandio AA, Fosu P, Asante D, Siaw A, Danquah FO, Korankye BA, Ntim-Amo G, Ansah S, Agbenyo W (2021) The impact of cooperative membership on fish farm households’ income: the case of Ghana. Sustainability 13(3):1059. 10.3390/su13031059

[CR7] BMZ (2023) Feminist Development Policy. Government of Germany: Federal Ministry for Economic Cooperation and Development. https://www.bmz.de/resource/blob/153806/bmz-strategy-feminist-development-policy.pdf

[CR8] BoP Innovation Center and FISH (2021) Identifying niches for women’s entrepreneurship in aquatic food chains: A methods package. Methodology guide. WorldFish, Penang, Malaysia. https://hdl.handle.net/20.500.12348/5069

[CR9] Boudet AMM (2013) On norms and agency: Conversations about gender equality with women and men in 20 countries. World Bank Publications

[CR10] Chete OB (2019) Gender and agricultural practice in developing countries: literature review. South Asian J Soc Stud Econom 5(2):1–11. 10.9734/sajsse/2019/v5i230142

[CR11] Cislaghi B, Heise L (2020) Gender norms and social norms: differences, similarities and why they matter in prevention science. Sociol Health Illn 42(2):407–422 31833073 10.1111/1467-9566.13008 PMC7028109

[CR12] Clucas IJ, Ward AR (1996) Post-harvest fisheries development: a guide to handling, preservation, processing and quality, pp ix+-443

[CR13] Cole SM, Kaminski AM, McDougall C, Kefi AS, Marinda PA, Maliko M, Mtonga J (2020) Gender accommodative versus transformative approaches: a comparative assessment within a post-harvest fish loss reduction intervention. Gend Technol Dev 24(1):48–65. 10.1080/09718524.2020.1729480

[CR14] Cole SM, Kantor P, Sarapura S, Rajaratnam S (2015) Gender-transformative approaches to address inequalities in food, nutrition and economic outcomes in aquatic agricultural systems. WorldFish

[CR15] Christopherson K, Yiadom A, Johnson J, Fernando F, Yazid H, Thiemann C (2022) Tackling legal impediments to women’s economic empowerment. International Monetary Fund

[CR16] Dolislager M, Reardon T, Arslan A, Fox L, Liverpool-Tasie S, Sauer C, Tschirley DL (2021) Youth and adult agrifood system employment in developing regions: rural (peri-urban to hinterland) vs. urban. J Develop Stud 57(4):571–593. 10.1080/00220388.2020.1808198

[CR17] Donkoh NE, Antwi D, Sarfo B (2020) Analysis of the role of women within the oil palm value chain in the Akyemansa District and Birim Central Municipality of the Eastern Region of Ghana. ADRRI J Agriculture Food Sci. 4(6 (4)):1–26. 10.55058/adrrijafs.v4i6(4).599

[CR18] Ehsan A, Klaas HS, Bastianen A, Spini D (2019) Social capital and health: a systematic review of systematic reviews. SSM-Population Health 8:100425. 10.1016/j.ssmph.2019.100425 31431915 10.1016/j.ssmph.2019.100425 PMC6580321

[CR19] Emmanuel O, Chinenye A, Oluwatobi A, Peter K (2014) Review of aquaculture production and management in Nigeria. Am J Experimental Agriculture 4(10):1137–1151. 10.9734/AJEA/2014/8082

[CR20] FAO (2022) The state of world fisheries and aquaculture 2022: towards blue transformation. Rome, FAO, 1–11. 10.4060/cc0461en

[CR21] FAO, IFAD and WFP. 2022. Guide to formulating gendered social norms indicators in the context of food security and nutrition Rome10.4060/cc0673en

[CR22] Farm Africa (2016) Market study of the aquaculture market in Kenya. In: Kenya Market-Led Aquaculture Programme (KMAP). Farm Africa, Kenya. https://www.farmafrica.org/wp-content/uploads/2024/07/study-of-the-kenyan-aquaculturemarket.pdf

[CR23] Farnworth CR, Lecoutere E, Galiè A, Van Campenhout B, Elias M, Ihalainen M, Roeven L, Bharati P, Valencia AMP, Crossland M, Vinceti B, Monterroso I (2023) Methodologies for researching feminization of agriculture: what do they tell us? Prog Dev Stud 23(3):294–316

[CR24] Farnworth CR, Jiggins J, Thomas EV (2016) Creating food futures: Trade, ethics and the environment. In: Creating Food Futures. Routledge, pp 1–7

[CR25] Fischer G, Patt N, Ochieng J, Mvungi H (2020) Participation in and gains from traditional vegetable value chains: a gendered analysis of perceptions of labour, income and expenditure in producers’ and traders’ households. Europ J Develop Res 32(4):1080–1104. 10.1057/s41287-020-00257-0

[CR26] Folorunso EA, Rahman MA, Sarfo I, Darko G, Olowe OS (2021) Catfish farming: a sustainability study at Eriwe fish farming village in southwest Nigeria. Aquaculture Int 29(2):827–435. 10.1007/s10499-021-00662-0

[CR27] Fox L, Pimhidzai O (2013) Different dreams, same bed: collecting, using, and interpreting employment statistics in Sub-Saharan Africa—the case of Uganda. World Bank Policy Research Working Paper, p 6436

[CR28] Galiè A, Kantor P (2016) From gender analysis to transforming gender norms: Using empowerment pathways to enhance gender equity and food security in Tanzania. In: Njuki J, Parkins J, Kaler A (eds) Transforming gender and food security in the global south. Routledge, pp 213–240

[CR29] Genschick S, Marinda P, Tembo G, Kaminski AM, Thilsted SH (2018) Fish consumption in urban Lusaka: the need for aquaculture to improve targeting of the poor. Aquaculture 492:280–289. https://www.researchgate.net/publication/324384068. Accessed 20 Mar 2023

[CR30] Githukia CM, Drexler SS, Obiero KO, Nyawanda BO, Odhiambo JA, Chesoli JW, Manyala JO (2020) Gender roles and constraints in the aquaculture value chain in Western Kenya. Afr J Agric Res 16(5):732–745. 10.5897/AJAR2020.14783

[CR31] Gopal N, Hapke HM, Kusakabe K, Rajaratnam S, Williams MJ (2020) Expanding the horizons for women in fisheries and aquaculture. Gend Technol Dev 24(1):1–9. 10.1080/09718524.2020.1736353

[CR32] Harrison E (2000) Men, women and work in rural Zambia. Eur J Dev Res 12:53–71

[CR33] Idiku FO, Ntui OE, Iyamah DA, Ochang DO (2022) Analysis of gender participation in fish production value chain in Akpabuyo, Cross River State Nigeria. World J Adv Res Rev 13(02):131–135. 10.30574/wjarr

[CR34] Idris I (2018) Barriers to women’s economic inclusion in Tanzania. Helpdesk Report

[CR35] Issa FO, Aderinoye-AbdulWahab S, Kagbu JH (2022) Assessment of aquaculture development programmes in Nigeria. J Agricultural Extension 26(1):10–17. 10.4314/jae.v26i1.2

[CR36] Jolly CM, Nyandat B, Yang Z, Ridler N, Matias F, Zhang Z., ... Menezes A (2023) Dynamics of aquaculture governance. J World Aquaculture Soc. 10.1111/jwas.12967

[CR37] Kiessel A (2022) Recentring Fair Trade in the movement for a just, inclusive and regenerative economy. Journal of Fair Trade 3(2):28–33

[CR38] Khor L, Bodunde OA, Wills R, Hanson L, Adeyemo OK, Aina OO, Alarape SA, Delamare-Deboutteville J, Chadag VM (2024) Understanding aquaculture biosecurity to improve catfish disease management in Ogun and Delta states, Nigeria. Aquaculture 584:740664

[CR39] Krause G, Billing S, Dennis J, Grant J, Fanning L, Miller M, Antonio P (2020) Visualizing the social in aquaculture: how social dimension components illustrate the effects of aquaculture across geographic scales. Mar Policy 118(2020):1–13. 10.1016/j.marpol.2020.103985

[CR40] Kruijssen F, McDougall CL, Van Asseldonk IJ (2018) Gender and aquaculture value chains: A review of key issues and implications for research. Aquaculture 493:328–337

[CR41] Kruijssen F, Danielsen K, Newton J, Braaten Y (2021) Gendered aquaculture value chain analysis and development: an analytical framework. CGIAR Research program on fish agri-food systems. Manual: FISH-2021-27. WorldFish

[CR42] Kruijssen F, Danielsen K, Newton J, Braaten Y (2022) Gendered aquaculture value chain analysis and development: An analytical framework

[CR43] Kumar G, Engle C, Tucker C (2018) Factors driving aquaculture technology adoption. J World Aquaculture Soc 49(3):447–476

[CR44] Kusakabe K (2003) Women's involvement in small-scale aquaculture in Northeast Thailand. Dev Pract 13(4):333–345. https://www.jstor.org/stable/4029658. Accessed 20 Mar

[CR45] Lawless S, Cohen P, McDougall C, Orirana G, Siota F, Doyle K (2019) Gender norms and relations: implications for agency in coastal livelihoods. Maritime Studies 18:347–358. 10.1007/s40152-01900147-0

[CR46] Leal Filho W, Setti AFF, Azeiteiro UM, Lokupitiya E, Donkor FK, Etim NN, Djekic I (2022) An overview of the interactions between food production and climate change. Sci Total Environ 838:156438 10.1016/j.scitotenv.2022.156438 35660578

[CR47] Lindström M (2020) A commentary on “The trouble with trust: time-series analysis of social capital, income inequality, and COVID-19 deaths in 84 countries.” Soc Sci Med 263:113386. 10.1016/j.socscimed.2020.113365 33036797 10.1016/j.socscimed.2020.113386 PMC7532747

[CR48] Manlosa AO, Schultner J, Dorresteijn I, Fischer J (2019) Leverage points for improving gender equality and human well-being in a smallholder farming context. Sustain Sci 14:529–541. 10.1007/s11625-018-0636-4

[CR49] Marcus R (2021) Gender, social norms, and women’s economic empowerment. Women's Economic Empowerment 126–153

[CR50] McDougall C, Badstue L, Mulema A, Fischer G, Najjar D, Pyburn R, ... Vos A (2021) Toward structural change: gender transformative approaches. Advancing Gender Equality through Agricultural and Environmental Research: Past, Present and Future, 365–402.

[CR51] Mensah-Bonsu A, Lartey NN, Kuwornu JK (2019) Gender-segregated analysis of the poultry value chain in Ghana. Gend Technol Dev 23(2):130–164. 10.1080/09718524.2019.1661611

[CR52] Morgan M, Terry G, Rajaratnam S, Pant J (2017) Socio-cultural dynamics shaping the potential of aquaculture to deliver development outcomes. Rev Aquac 9(4):317–325. 10.1111/raq.12137

[CR53] Mwongera C, Shikuku KM, Twyman J, Läderach P, Ampaire E, Van Asten P, Twomlow S, Winowiecki LA (2017) Climate smart agriculture rapid appraisal (CSA-RA): a tool for prioritizing context-specific climate smart agriculture technologies. Agric Syst 151:192–203. 10.1016/j.agsy.2016.05.009

[CR54] Nasr-Allah A, Gasparatos A, Karanja A, Dompreh EB, Murphy S, Rossignoli CM, ... Charo-Karisa H (2020) Employment generation in the Egyptian aquaculture value chain: implications for meeting the sustainable development goals (SDGs). Aquaculture. 520l:734940. 10.1016/j.aquaculture.2020.734940

[CR55] Ndanga LZB, Quagrainie KK, Dennis JH (2013) Economically feasible options for increased women participation in Kenyan aquaculture value chain. Aquaculture 415:183–190. 10.1016/j.aquaculture.2013.08.012

[CR56] Nussbaum MC (2003) Capabilities as fundamental entitlements: Sen and social justice. Fem Econ 9(2–3):33–59. 10.1080/1354570022000077926

[CR57] Nwabueze TU (2010) Basic steps in adapting response surface methodology as mathematical modelling for bioprocess optimisation in the food systems. Int J Food Sci Technol 45(9):1768–1776

[CR58] OECD (2021) Man enough? Measuring masculine norms to promote women’s empowerment, social institutions and gender index. OECD Publishing, Paris. 10.1787/6ffd1936-en

[CR59] Olaoye OJ, Odebiyi OC (2011) Economic viability for the use of microfinance bank loanon aquaculture development in Ogun State, Nigeria. Int J Fish Aquac 3(4):70–77

[CR60] Olaoye OJ, Ashley-Dejo SS, Fakoya EO, Ikeweinwe NB, Alegbeleye WO, Ashaolu FO, Adelaja OA (2013) Assessment of socio-economic analysis of fish farming in Oyo State, Nigeria. Global J Sci Frontier Res Agriculture Vet. 13(9):45–55. http://journalofagriculture.org/index.php/GJSFR/article/viewFile/105/105. Accessed 20 Mar 2023

[CR61] Olufayo MO (2012) The Gender roles of women in aquaculture and food security in Nigeria. Visible Possibilities: The economics of sustainable fisheries, aquaculture and seafood Trade. In: Shriver AL (ed) Proceedings of the sixteenth biennial conference of the international institute of fisheries economics and trade, July 16-20, Dar es Salaam, Tanzania. International Institute of Fisheries Economics and Trade (IIFET), Corvallis

[CR62] Omananyi M (2021) Involvement of small and medium scale enterprises in culture fish value chain in Niger State, Nigeria (Doctoral dissertation). Federal University of Technology Minna

[CR63] Omeje J, Achike A, Sule A, Arene C (2021) gender roles and economic differentials in aquaculture of Kainji Lake Basin, Nigeria. Research on World Agricultural Economy. 02(02):1–10. http://ojs.nassg.org/index.php/rwae. Accessed 20 Mar 2023

[CR64] Omitoyin SA, Fawehinmi OA, Pomary AB (2011) Gender participation in aquaculture in Lagos State, Nigeria. J Gend Stud3–4.https://www.researchgate.net/publication/349850365_GENDER_PARTICIPATION_IN_AQUACULTURE_IN_LAGOS_STATE_NIGERIA. Accessed 20 Mar 2023

[CR65] Pavo RR, Digal LN (2016) Women’s space in the fish port Tambler Complex and the value-chain nodes of the fishing industry in General Santos City, Philippines. Gender in Aquaculture and Fisheries: Engendering Security in Fisheries and Aquaculture, 33.

[CR66] Rolleri LA (2013) Can gender norms change. Research facts and findings. ACT for Youth, Ithaca, NY

[CR67] Quaye W, Dowuona S, Okai M, Dziedzoave N (2016) Gender dimensions of decision-making on production assets and challenges facing women. Dev Pract 26(1):77–90. 10.1080/09614524.2016.1112364

[CR68] Ramirez PJ, Narvaez TA, Santos-Ramirez EJ (2020) Gender-inclusive value chains: the case of seaweed farming in Zamboanga Peninsula, Philippines. Gend Technol Dev 24(1):110–130. 10.1080/09718524.2020.1728810

[CR69] Rietveld AM, Farnworth CR, Shijagurumayum M, Meentzen A, Voss RC, Morahan G, López D E (2023) An evidence synthesis of gender norms in agrifood systems: Pathways towards improved women’s economic resilience to climate change

[CR70] Sakoda JM (1981) A generalized index of dissimilarity. Demography 18(2):245–250 7227588

[CR71] Sandefur RL, Laumann EO (1998) A paradigm for social capital. Ration Soc 10(4):481–501. 10.1177/104346398010004005

[CR72] Sasa S, Adebayo E, Maurice D (2022) Constraints to women participation in agriculture and economic development in Nigeria: a review. Constraints. 8(5). https://www.ijaar.org/articles/v8n5/sms/ijaar-v8n5-May22-p8508.pdf

[CR73] Sen Amartya (1995) Gender inequality and theories of justice 1. 10.1093/0198289642.003.0011.

[CR74] Shailaja E, Madeleine E (2008) Gender education and equality in a global context : conceptual frameworks and policy perspectives Gender Education and Equality in a Global Context. Routledge, London. 10.1080/09540250802211184

[CR75] Subasinghe RP, Siriwardena SN, Byrd KA, Chan C, Dizyee K, Shikuku KM Tran N, Adegoke A, Adeleke L, Anastasiou K, Beveridge M (2021) Nigeria fish futures. Aquaculture in Nigeria: increasing income, diversifying diets and empowering women. Report of the scoping study. https://digitalarchive.worldfishcenter.org/handle/20.500.12348/4951. Accessed 20 Mar 2023

[CR76] Szymkowiak M (2020) Genderizing fisheries: assessing over thirty years of women’s participation in Alaska fisheries. Mar Policy 115:103846. 10.1016/j.marpol.2020.103846

[CR77] Tikadar KK, Islam MJ, Saha SM, Alam MM, Barman SK, Rahman MA (2022) Livelihood status of small-scale fishermen and determinants of their income: insights from north-eastern floodplains of Bangladesh. Geography Sustain 3(3):204–213. 10.1016/j.geosus.2022.06.002

[CR78] Tran N, Chu L, Chan CY, Genschick S, Phillips MJ, Kefi AS (2019) Fish supply and demand for food security in Sub-Saharan Africa: an analysis of the Zambian fish sector. Marine Policy. 99(September 2018):343–350. 10.1016/j.marpol.2018.11.009

[CR79] UN DESA (2023) The Sustainable Development Goals Report 2023: Special Edition - July 2023. New York, USA: UN DESA. © UN DESA. https://unstats.un.org/sdgs/report/2023/

[CR80] Weeratunge N, Snyder KA, Choo PS (2010) Gleaner, fisher, trader, processor: understanding gendered employment in fisheries and aquaculture. Fish 11(4):405–420. 10.1111/j.1467-2979.2010.00368.x

[CR81] Welzel C, Inglehart R, Deutsch F (2005) Social capital, voluntary associations and collective action: which aspects of social capital have the greatest ‘civic ‘payoff? J Civ Soc 1(2):121–146. 10.1080/17448680500337475

[CR82] Zidrou C, Kleisiaris C, Adamakidou T (2021) Associations between disability in activities of daily living and social capital aspects among older adults: a scoping review. Journal of Frailty, Sarcopenia and Falls 6(3):119. 10.22540/2FJFSF-06-119 34557611 10.22540/JFSF-06-119 PMC8419853

